# Therapeutic efficacy of dihydroartemisinin-piperaquine and artesunate-pyronaridine combinations in the treatment of uncomplicated *Plasmodium falciparum* malaria in Ghana, 2023

**DOI:** 10.3389/fpubh.2025.1715777

**Published:** 2026-01-05

**Authors:** Benjamin Abuaku, Paul Boateng, Nana Yaw Peprah, Alexander Asamoah, Nancy Odurowah Duah-Quashie, Sena Adzoa Matrevi, Eunice Obeng Amoako, Neils Quashie, Felicia Owusu-Antwi, Kwadwo Ansah Koram, Keziah Laurencia Malm

**Affiliations:** 1 Department of Epidemiology, Noguchi Memorial Institute for Medical Research, College of Health Sciences, University of Ghana, Accra, Ghana; 2 National Malaria Elimination Program, Public Health Division, Ghana Health Service, Accra, Ghana; 3 Centre for Tropical Clinical Pharmacology and Therapeutics, University of Ghana Medical School, Accra, Ghana; 4 World Health Organization, Country Office, Accra, Ghana

**Keywords:** artesunate-pyronaridine, dihydroartemisinin-piperaquine, efficacy, Ghana, treatment, uncomplicated malaria

## Abstract

**Introduction:**

Malaria case management in Ghana is a key intervention within the national malaria elimination agenda. Currently, artesunate-amodiaquine (AS-AQ), artemetherlumefantrine (AL), and artesunate-pyronaridine (AP) are the first-line medicines for treating uncomplicated malaria whilst dihydroartemisinin-piperaquine (DHAP) is the second-line medicine.

**Methods:**

This was a one-arm prospective evaluation of the clinical, parasitological and haematological responses of children (6 months to 9 years old) treated with DHAP in five (5) sentinel sites and AP in four (4) sentinel sites between August and December, 2023 using the WHO protocol on surveillance of antimalarial drug efficacy. The DHAP study was a follow-up to the baseline conducted in 2020/2021, and the AP study was to provide baseline data after its introduction in 2022. The 3/3 PCR-genotyping approach distinguished between reinfection and recrudescence using merozoite surface protein 1 (*msp1*)-specific primers: RO33, MAD20, K1; merozoite protein 2 (*msp2*)-specific primers: IC 3D7 and FC; and glutamate-rich protein (*glurp*).

**Results:**

PCR-uncorrected cure rates on day-42 ranged between 98.8% (95% CI: 93.2-100) and 100% for DHAP with an overall PCR-uncorrected cure rate of 99.2% (95% CI: 97.6-99.8); and between 85.0% (95% CI: 70.2-94.3) and 100% for AP with an overall PCR-uncorrected cure rate of 96.2% (95% CI: 92.9-98.0). PCR-corrected cure rates on day-42 was 100% in all DHAP sites; and ranged between 91.9% (95% CI: 78.1-98.3) and 100% for AP with an overall PCR-corrected cure rate of 97.3% (95% CI: 94.3-98.8). Day-3 parasitemia was prevalent in only one (1) DHAP site (1/60 – 1.7%) and one (1) AP site (1/65 – 1.5%) with an overall prevalence of 0.3% for DHAP and 0.4% for AP. Neither treatment with DHAP nor AP resulted in an early treatment failure (ETF).

**Conclusion:**

We conclude that the therapeutic efficacy level of DHAP has remained high (>90%) since the baseline study in 2020/2021. Also, the baseline efficacy level of AP is high (>90%) warranting the use of both DHAP and AP in the treatment of uncomplicated malaria in the country.

## 1 Introduction

Malaria remains one of the major public health problems in the world affecting 263 million individuals in 2023 with an incidence of 60.4 cases per 1,000 population at risk (an increase over the 2022 incidence of 58.6 cases per 1,000 population at risk), and sub-Sahara Africa accounting for about 94% of the reported global malaria cases and 95% of the reported 597,000 global malaria deaths (1). In Ghana, a total of 5.2 million confirmed malaria cases and 151 malaria-related deaths were reported in 2022 (2). The number of malaria cases reported in 2022 accounted for approximately 20% of all outpatient attendance and 22% of all admissions (2). Malaria, therefore, remains one of the leading causes of morbidity and mortality in Ghana with 95.8–100% of infections attributed to *Plasmodium falciparum* (2).

Ghana’s malaria elimination agenda, launched in 2024, has case management as a key intervention within all the different transmission zones across the country (2). Malaria case management involves early diagnosis and prompt effective treatment using artemisinin-based combination therapy (ACT) (3). Artemisinin-based combination therapy was introduced in Ghana in 2005, when artesunate-amodiaquine (AS-AQ) combination was deployed across the country to replace chloroquine (CHQ) as first-line medicine for treating uncomplicated malaria (4). Dihydroartemisinin-piperaquine (DHAP) and artemether-lumefantrine (AL) were introduced in 2008 as alternate ACTs for the treatment of uncomplicated malaria (5). In 2020, AS-AQ and AL remained first-line ACTs whilst DHAP was made a second-line ACT for the treatment of uncomplicated malaria (6). In 2022, artesunate-pyronaridine (AP) was introduced as an alternate first-line ACT (7).

Currently, Ghana has three first-line ACTs for the treatment of uncomplicated malaria: AS-AQ, AL, and AP whilst DHAP remains the second-line ACT for the treatment of uncomplicated malaria (7, 8). To monitor the therapeutic efficacy of the first-line and second-line ACTs for uncomplicated malaria, Ghana continues to implement a surveillance system across the country aimed at tracking the efficacy of these drugs to support timely decisions on malaria treatment policy and case management guidelines. We report the therapeutic efficacy of AP and DHAP studied in 2023 at sentinel sites across the country using the 2009 World Health Organization (WHO) protocol on surveillance of antimalarial drug efficacy (9). This is the first efficacy report for AP since its introduction in 2022 and the second for DHAP since 2020 (10).

## 2 Materials and methods

### 2.1 Study sites

**Figure 1:**
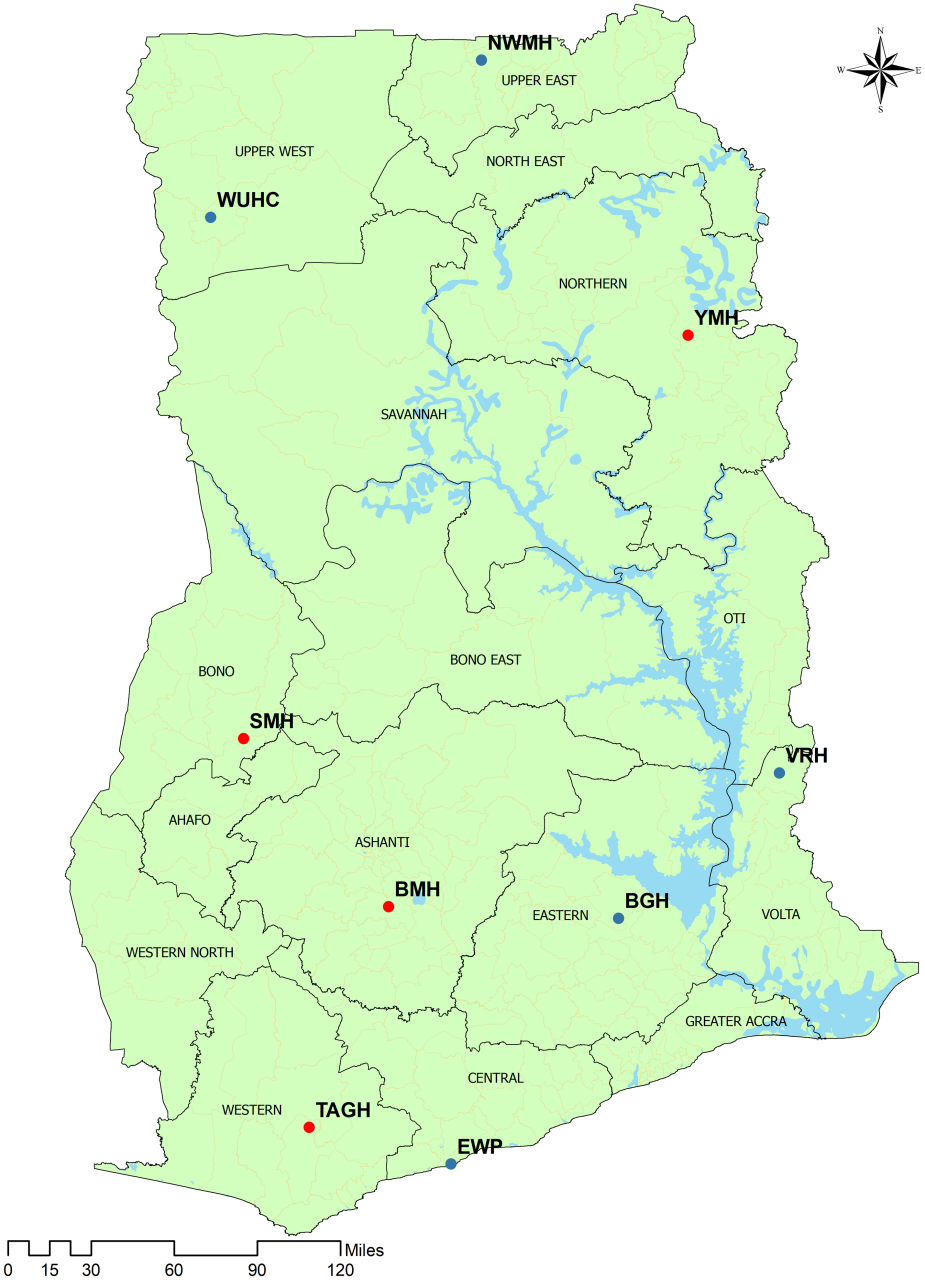
Map of Ghana showing nine therapeutic efficacy study (TES) sites. The blue and red dots are sites that studied DHAP and AP, respectively. WUHC, Wa Urban Health Centre; NWMH, Navrongo War Memorial Hospital; VRH, Volta Regional Hospital; BGH, Begoro Government Hospital; EWP, Ewim Polyclinic; TAGH, Tarkwa Apinto Government Hospital; YMH, Yendi Municipal Hospital; BMH, Bekwai Municipal Hospital; SMH, Sunyani Municipal Hospital.

The study was conducted in nine of ten sentinel sites established in 2005 to monitor the therapeutic efficacy of first-line and second-line antimalaria medicines in Ghana. Five sites studied DHAP whilst four sites studied AP. The sites that studied DHAP were Wa Urban Health Centre (WUHC), and Navrongo War Memorial Hospital (NWMH), both located in the savannah zone; Begoro Government Hospital (BGH) and Volta Regional Hospital (VRH), formerly Hohoe Municipal Hospital, located in the forest zone; and Ewim polyclinic (EWP), Cape-Coast, located in the coastal zone. The four sites that studied AP were Yendi Municipal Hospital (YMH) located in the savannah zone; and Sunyani Municipal Hospital (SMH), Bekwai Municipal Hospital (BMH), and Tarkwa Apinto Government Hospital (TAGH), all located within the forest zone (Figure 1). These sites have been described elsewhere (11). In 2020/2021 DHAP was studied in NWMH, VRH, and EWP. The Day-42 PCR-corrected cure rates at that time were 90.3% in NWMH and 100% in both VRH and EWP (10).

### 2.2 Study design

The study was a one-arm prospective evaluation of the clinical, parasitological and haematological responses of children treated with AP in four sentinel sites and with DHAP in five sentinel sites using the WHO protocol on surveillance of antimalarial drug efficacy (9).

### 2.3 Patient enrolment, treatment and follow-up

Children were enrolled into the study when they met the following inclusion criteria: aged between 6 months and 9 years, a history of fever within the past 2–3 days or an axillary temperature ≥37.5 °C, mono-infection with *Plasmodium falciparum* (*Pf*), asexual *Pf* density of between 1,000 and 250,000 per μL, ability to swallow oral medications, and parent/guardian giving consent and willing to comply with study protocol and visit schedule for the duration of the study. Children were excluded from the study when they met the following criteria: presence of general danger signs or signs of severe malaria, mixed or mono-infection with another *Plasmodium* species, severe acute malnutrition, hypersensitivity to the antimalarial drug being tested (DHAP or AP), or parent/guardian refusing to give consent.

All treatments were given by a study nurse, and each treatment was based on the child’s weight as per the manufacturer’s instructions (Tables 1, 2). Each child was observed for 30 min after drug administration to ascertain retention of the medicine administered. Children who vomited within the 30 min of observation had their treatment repeated with the same dose and observed for another 30 min. Children with repeated vomiting were excluded from the study and given parenteral artesunate as per national treatment guidelines for severe malaria (8). Details of the DHAP used are: 20 mg/160 mg and 40 mg/320 mg of D-ARTEPP® (products of Guilin Pharmaceutical Company Limited, Guangxi, China) whilst details of the AP used are: 20 mg/60 mg and 60 mg/180 mg of PYRAMAX® (products of Shin Poong Pharmaceutical Company Limited, Seoul, South Korea).

**Table 1:** Treatment doses for dihydroartemisinin-piperaquine (DHAP) combination.

Weight (kg)	Dihydroartemisinin-piperaquine base	Total dose	Day		
			0	1	2
From 5 kg to less than 8 kg (≥5 kg to <8 kg)	20 mg/160 mg	3 tablets	1	1	1
From 8 kg to less than 11 kg (≥8 kg to <11 kg)	20 mg/160 mg	4½ tablets	1 ½	1 ½	1 ½
From 11 kg to less than 17 kg (≥11 kg to <17 kg)	40 mg/320 mg	3 tablets	1	1	1
From 17 kg to less than 25 kg (≥17 kg to <25 kg)	40 mg/320 mg	4½ tablets	1 ½	1 ½	1 ½
From 25 kg to less than 36 kg (≥25 kg to <36 kg)	40 mg/320 mg	6 tablets	2	2	2

**Table 2:** Treatment doses for artesunate-pyronaridine (AP) combination.

Weight (kg)	Artesunate/pyronaridine base	Total dose	Day		
			0	1	2
From 5 kg to less than 8 kg (≥5 kg to <8 kg)	20 mg/60 mg	3 sachets	1	1	1
From 8 kg to less than 15 kg (≥8 kg to <15 kg)	20 mg/60 mg	6 sachets	2	2	2
From 15 kg to less than 20 kg (≥15 kg to <20 kg)	20 mg/60 mg	9 sachets	3	3	3
From 20 kg to less than 24 kg (≥20 kg to <24 kg)	60 mg/180 mg	3 tablets	1	1	1
From 24 kg to less than 45 kg (≥24 kg to <45 kg)	60 mg/180 mg	6 tablets	2	2	2

Day of enrolment was classified as Day 0, and the follow-up period for both drugs covered 42 days. The schedule of activities was: clinical assessments (Days 0, 1, 2, 3, 7, 14, 21, 28, 35, 42, and any unscheduled visit within the 42-day follow-up period); drug administration (Day 0–Day 2); parasitological examination (Days 0, 2, 3, 7, 14, 21, 28, 35, 42, and any unscheduled visit within the 42-day follow-up period); and haemoglobin level assessments (Days 0, 28, and 42).

All malaria blood slides read by site microscopists were examined by a WHO-certified (level 1) microscopist. Slides with discordant readings (in terms of presence or absence of asexual/sexual parasites, species identification, and Day 0 asexual parasite count meeting the inclusion criterion of 1,000–250,000 per μL blood) were re-examined by a third WHO-certified microscopist. Parasite recrudescence was distinguished from re-infection by PCR genotyping using merozoite surface protein 1 (msp1)-specific primers: RO33, MAD20, K1; merozoite protein 2 (msp2)-specific primers: IC 3D7 and FC; and glutamate-rich protein (glurp). A classification of recrudescence was made when three loci matched (3/3 approach) as recommended (Supplementary Table S1) (12, 13).

### 2.4 Data analysis

A minimum sample of 88 children was estimated for each study site based on assumed PCR-corrected treatment failure rate of 5% at 95% confidence level, 5% precision, and 20% loss to follow-up. The WHO Excel® template for therapeutic efficacy tests (9) was used to capture data for each child. The primary study outcomes were presence of parasitaemia on Day 3 of follow-up and PCR-uncorrected and PCR-corrected efficacy outcomes on Day 28 and Day 42 using both per protocol and Kaplan Meier survival analyses. Treatment outcomes followed WHO classifications as follows: early treatment failure (ETF), late parasitological failure (LPF), late clinical failure (LCF), and adequate clinical and parasitological response (ACPR) (9). Secondary study outcomes were fever (axillary temperature ≥ 37.5 °C) clearance, parasite clearance, changes in mean haemoglobin levels on Day 28 and Day 42 post-treatment with Day 0 as baseline, and prevalence of adverse events. Proportions were compared using Chi-square and Fisher’s exact tests whilst means were compared using Student’s *t*-test/ANOVA (significant at *p* < 0.05).

## 3 Results

### 3.1 Patient characteristics

**Figure 2:**
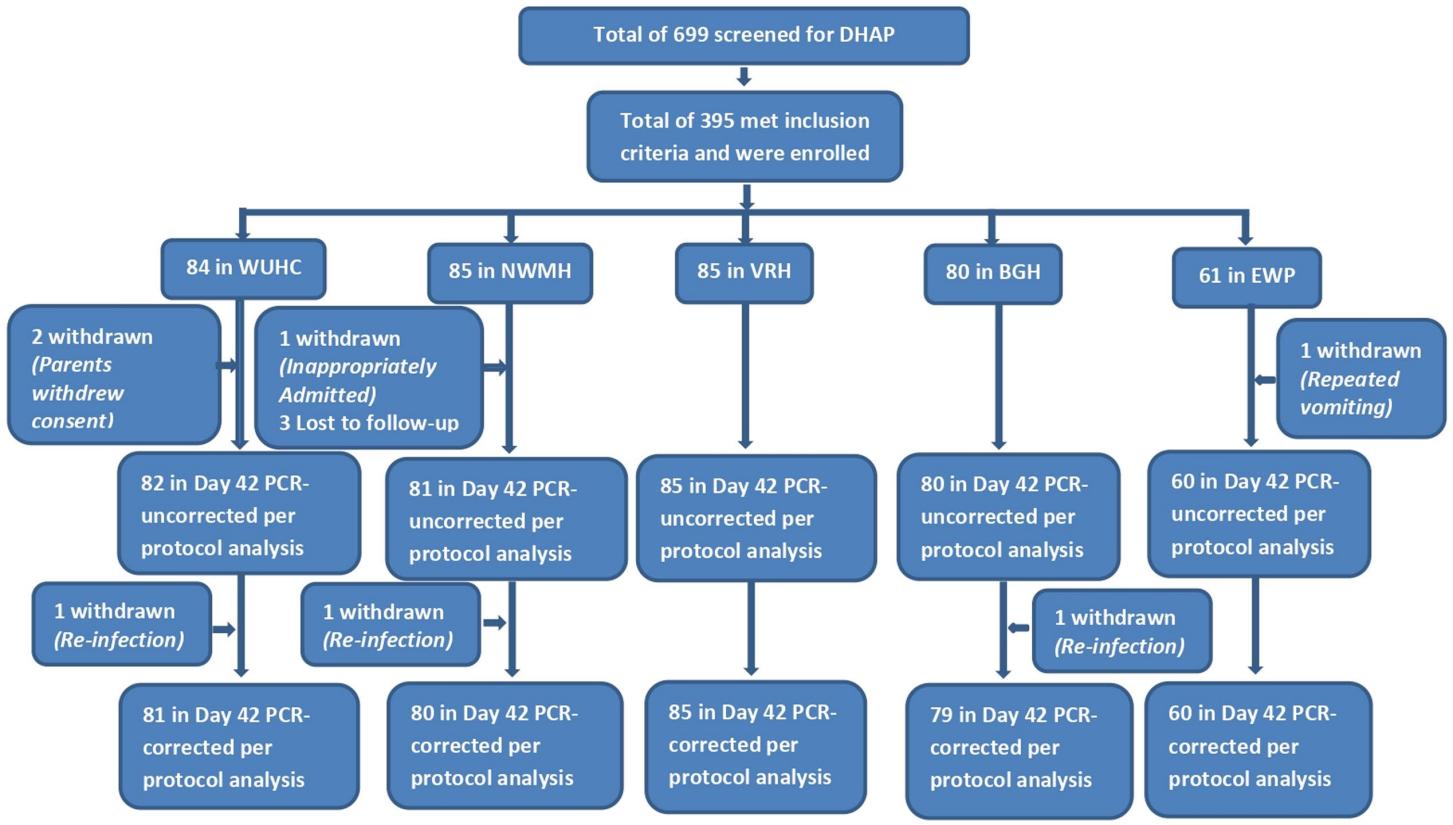
Flow chart showing number of children screened, enrolled, and included in per-protocol analysis following treatment with dihydroartemisinin-piperaquine (DHAP). WUHC, Wa Urban Health Centre; NWMH, Navrongo War Memorial Hospital; VRH, Volta Regional Hospital; BGH, Begoro Government Hospital; EWP, Ewim Polyclinic.

**Figure 3:**
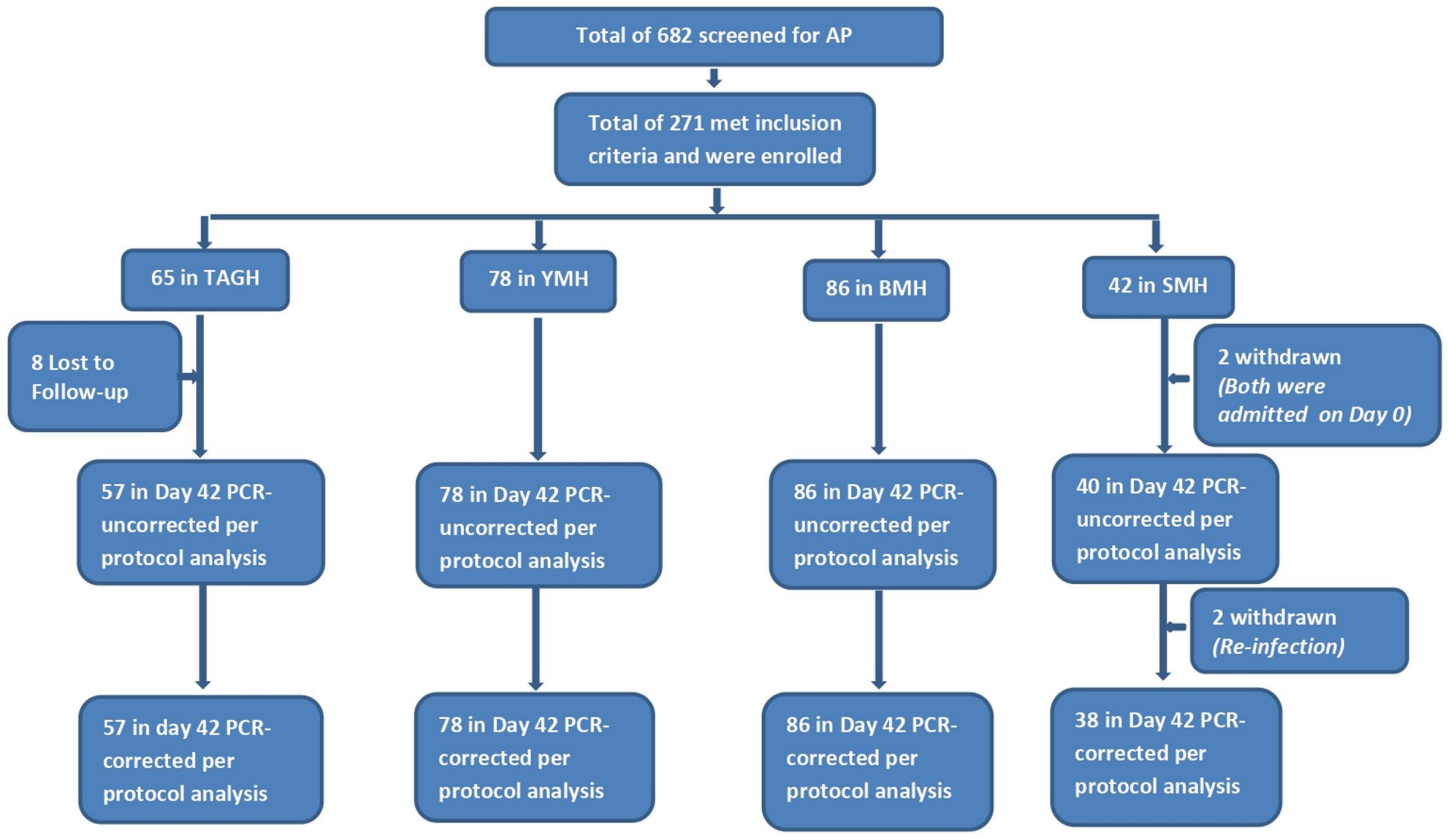
Flow chart showing number of children screened, enrolled, and included in per-protocol analysis following treatment with artesunate-pyronaridine (AP). TAGH, Tarkwa Apinto Government Hospital; YMH, Yendi Municipal Hospital; BMH, Bekwai Municipal Hospital; SMH, Sunyani Municipal Hospital.

A total of 699 children in the five sites studying DHAP and 682 children in the four sites studying AP were screened during the period of August–December 2023. Out of the number of children screened in the DHAP sites, 395 met the inclusion criteria and were subsequently enrolled whilst 271 of the total number screened in the AP sites were enrolled into the study (Figures 2, 3). Among children enrolled in the DHAP sites, the proportion of males in each site was higher than females; mean age in years ranged between 4.7 ± 2.7 in VRH and 5.5 ± 2.2 in NWMH (*p* = 0.197); majority of children in all sites were aged 5–9 years; mean weight in kilograms ranged between 17.3 ± 6.1 in BGH and 19.0 ± 5.5 in NWMH (*p* = 0.506); and mean axillary temperature ranged between 38.0 °C (± 0.9) in WUHC and 38.7 °C (±0.6) in VRH (*p* < 0.001) (Table 3). Geometric mean parasite density ranged between 19,603/μl in VRH and 29,318/μl in BGH (p < 0.001) whilst mean haemoglobin level ranged between 9.3 g/dL (±1.7) in EWP and 10.7 g/dL (± 1.5) in VRH (*p* < 0.001) (Table 3). The overall prevalence of gametocytaemia among children enrolled in the DHAP sites was 1.3% (5/395), and none of the children had a history of previous intake of an antimalarial before visiting the clinic.

**Table 3:** Background characteristics of patients enrolled in DHAP sites.

Characteristic	Total (*N* = 395)	Sentinel site					*p*-value
		WUHC (*N* = 84)	NWMH (*N* = 85)	VRH (*N* = 85)	BGH (*N* = 80)	EWP (*N* = 61)	
Gender							
Male	243 (61.5%)	46 (54.8%)	54 (63.5%)	52 (61.2%)	52 (65.0%)	40 (65.6%)	
Female	152 (38.5%)	38 (45.2%)	31 (36.5%)	33 (38.8%)	29 (35.0%)	21 (34.4%)	0.630
Mean age in years (SD)	5.0 (2.6)	4.8 (2.6)	5.5 (2.2)	4.7 (2.7)	4.9 (2.7)	5.3 (2.7)	0.197
Age group (years)							
<5	164 (41.5%)	41 (48.8%)	26 (30.6%)	38 (44.7%)	36 (45.0%)	23 (37.7%)	
5–9	231 (58.5%)	43 (51.2%)	59 (69.4%)	47 (55.3%)	44 (55.0%)	38 (62.3%)	0.083
Weight (kg)							
Mean weight (SD)	18.4 (6.4)	18.3 (7.1)	19.0 (5.5)	18.6 (6.9)	17.3 (6.1)	18.6 (6.4)	0.506
Range (min, max)	5.1, 36.0	6.5, 36.0	7.2, 33.0	8.0, 34.0	6.4, 36.0	5.1, 28.0	
Axillary temperature (°C)							
Mean temperature (SD)	38.3 (0.9)	38.0 (0.9)	38.4 (0.9)	38.7 (0.6)	38.1 (0.8)	38.4 (0.9)	<0.001
Range (min, max)	36.0, 40.4	36.2, 40.0	36.5, 40.4	37.0, 40.3	36.5, 40.0	36.0, 40.0	
Parasitemia/μL							
Geometric mean	23,669	29,082	7,356	19,603	68,494	29,318	<0.001
Range (min, max)	1,077, 249,465	1,094, 215,445	1,077, 1,963,370	1940, 108,216	12,985, 248,423	1,162, 249,465	
Haemoglobin level (g/dL)							
Mean (SD)	10.2 (1.5)	10.1 (1.5)	10.2 (1.4)	10.7 (1.5)	10.6 (1.3)	9.3 (1.7)	<0.001
Range (min, max)	5.6, 13.9	5.6, 13.5	6.9, 12.9	5.6, 13.8	7.1, 13.5	5.9, 13.9	

SD, standard deviation; DHAP, dihydroartemisinin-piperaquine; WUHC, Wa Urban Health Centre; NWMH, Navrongo War Memorial Hospital; VRH, Volta Regional Hospital; BGH, Begoro Government Hospital; EWP, Ewim Polyclinic.

Generally, the male/female ratio of children enrolled in the AP sites was almost 1:1 (141/130). Whereas the majority of children enrolled in TAGH were female (52.3%), the majority of children enrolled in YMH (55.1%) and SMH (57.1%) were male, though differences were not statistically significant (*p* = 0.716) (Table 4). Mean age in years ranged between 2.6 (±2.7) in BMH and 4.8 (±2.4) in SMH (*p* < 0.001). Majority of children in YMH (64.1%) and BMH (75.6%) were aged <5 years compared with the 47.7% in TAGH and 45.2% in SMH (*p* = 0.001) (Table 4). Mean weight in kilograms ranged between 16.2 (±7.5) in BMH and 20.4 (±9.8) in SMH (*p* = 0.019); mean axillary temperature ranged between 37.2 °C (±0.2) in YMH and 38.4 °C (±0.8) in TAGH (*p* < 0.001); geometric mean parasite density ranged between 14,678/μl in TAGH and 77,350/μl in NWMH (*p* < 0.001); mean haemoglobin levels ranged between 10.4 g/dL (±1.6) in TAGH and 10.6 g/dL (±0.9), 10.6 g/dL (±1.1), 10.6 g/dL (±1.4) in YMH, BMH, and SMH, respectively (*p* = 0.831) (Table 4). The overall prevalence of gametocytaemia among children enrolled in the AP sites was 0.7% (2/271), and none of the children had a history of previous intake of an antimalarial before visiting the clinic.

**Table 4:** Background characteristics of patients enrolled in AP sites.

Characteristic	Total (*N* = 271)	Sentinel site				*p*-value
		TAGH (*N* = 65)	YMH (*N* = 78)	BMH (*N* = 86)	SMH (*N* = 42)	
Gender						
Male	141 (52.0%)	31. (47.7%)	43 (55.1%)	43 (50.0%)	24 (57.1%)	
Female	130 (48.0%)	34 (52.3%)	35 (44.9%)	43 (50.0%)	18 (42.9%)	0.716
Mean age in years (SD)	3.8 (2.6)	4.5 (2.4)	3.8 (2.2)	2.6 (2.7)	4.8 (2.4)	<0.001
Age group (years)						
<5	165 (60.9%)	31 (47.7%)	50 (64.1%)	65 (75.6%)	19 (45.2%)	
5–9	106 (39.1%)	34 (52.3%)	28 (35.9%)	21 (24.4%)	23 (54.8%)	0.001
Weight (kg)						
Mean weight (SD)	17.5 (7.9)	18.5 (6.6)	16.6 (7.9)	16.2 (7.5)	20.4 (9.8)	0.019
Range (min, max)	6.0, 45.0	8.0, 37.0	7.0, 45.0	6.0, 38.0	7.8, 45.0	
Axillary temperature (°C)						
Mean temperature (SD)	37.9 (0.7)	38.4 (0.8)	37.2 (0.2)	38.1 (0.6)	38.1 (0.6)	<0.001
Range (min, max)	35.9, 40.3	36.9, 40.0	36.2, 37.9	35.9, 39.5	36.6, 40.3	
Parasitemia/μL						
Geometric mean	26,174	14,678	77,350	16,108	23,145	<0.001
Range (min, max)	1,301, 237,143	1,320, 237,143	1,593, 197,156	1,301, 214,542	1,500, 218,666	
Haemoglobin level (g/dL)						
Mean (SD)	10.6 (1.2)	10.4 (1.6)	10.6 (0.9)	10.6 (1.1)	10.6 (1.4)	0.831
Range (min, max)	6.2, 14.0	6.2, 13.2	8.9, 12.6	8.2, 13.5	6.4, 14.0	

SD, standard deviation; AP, artesunate-pyronaridine; TAGH, Tarkwa Apinto Government Hospital; YMH, Yendi Municipal Hospital; BMH, Bekwai Municipal Hospital; SMH, Sunyani Municipal Hospital.

### 3.2 Primary study outcomes

Day 3 parasitaemia was prevalent in only one DHAP site (EWP) and one AP site (TAGH) with site-specific prevalence of 1.7% (1/60) and 1.5% (1/65), respectively, yielding a national prevalence of 0.3% (95% CI: 0.01–1.43) or 1/388 for DHAP and 0.4% (95% CI: 0.01–2.1) or 1/269 for AP. There were no early treatment failures (ETF) among patients treated with either DHAP or AP (Supplementary Tables S2, S3).

**Figure 4:**
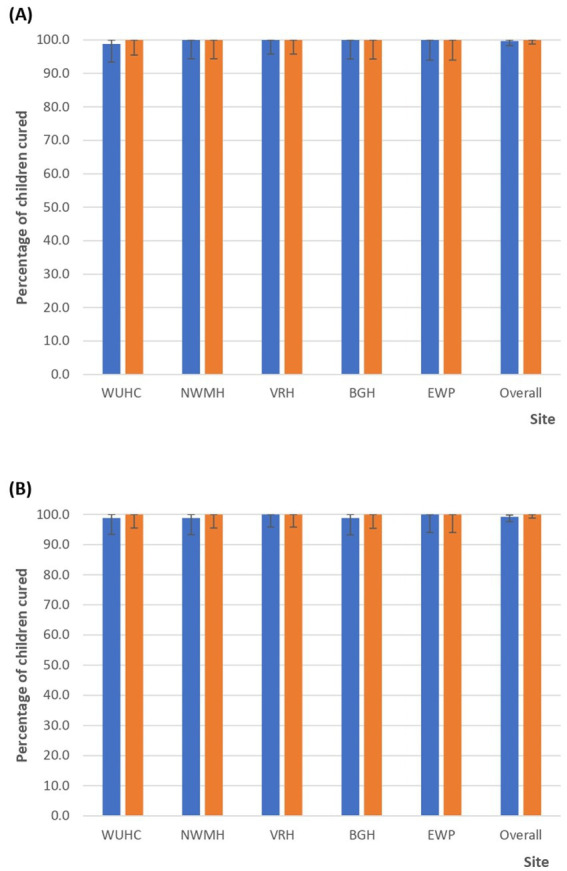
**(A)** Per protocol PCR-uncorrected and PCR-corrected cure rates on Day 28 following treatment with dihydroartemisinin-piperaquine (DHAP). Blue bars are PCR-uncorrected cure rates whilst orange bars are PCR-corrected cure rates. WUHC, Wa Urban Health Centre; NWMH, Navrongo War Memorial Hospital; VRH, Volta Regional Hospital; BGH, Begoro Government Hospital; EWP, Ewim Polyclinic. **(B)** Per protocol PCR-uncorrected and PCR-corrected cure rates on Day 42 following treatment with dihydroartemisinin-piperaquine (DHAP). Blue bars are PCR-uncorrected cure rates whilst orange bars are PCR-corrected cure rates. WUHC, Wa Urban Health Centre; NWMH, Navrongo War Memorial Hospital; VRH, Volta Regional Hospital; BGH, Begoro Government Hospital; EWP, Ewim Polyclinic.

Per protocol analyses on day 28 showed PCR-uncorrected cure rates of 100% in four of the five sites that studied DHAP: NWMH, VRH, BGH, and EWP. The only treatment failure among children who received DHAP occurred in WUHC, and was confirmed as re-infection yielding a Day-28 PCR-corrected cure rate of 100% in all DHAP sites (Figure 4A). PCR-uncorrected cure rates on Day 42, following treatment with DHAP, were 100% in two sites (VRH and EWP), 98.8% (95% CI: 93.4–100) in WUHC, 98.8% (95% CI: 93.3–100) in NWMH, and 98.8% (95% CI: 93.2–100) in BGH yielding an overall PCR-uncorrected cure rate of 99.2% (95% CI: 97.6–99.8). The treatment failures were all confirmed to be re-infections yielding a Day 42 PCR-corrected cure rate of 100% in all sites, and subsequently an overall Day-42 PCR-corrected cure rate of 100% (Figure 4B).

**Figure 5:**
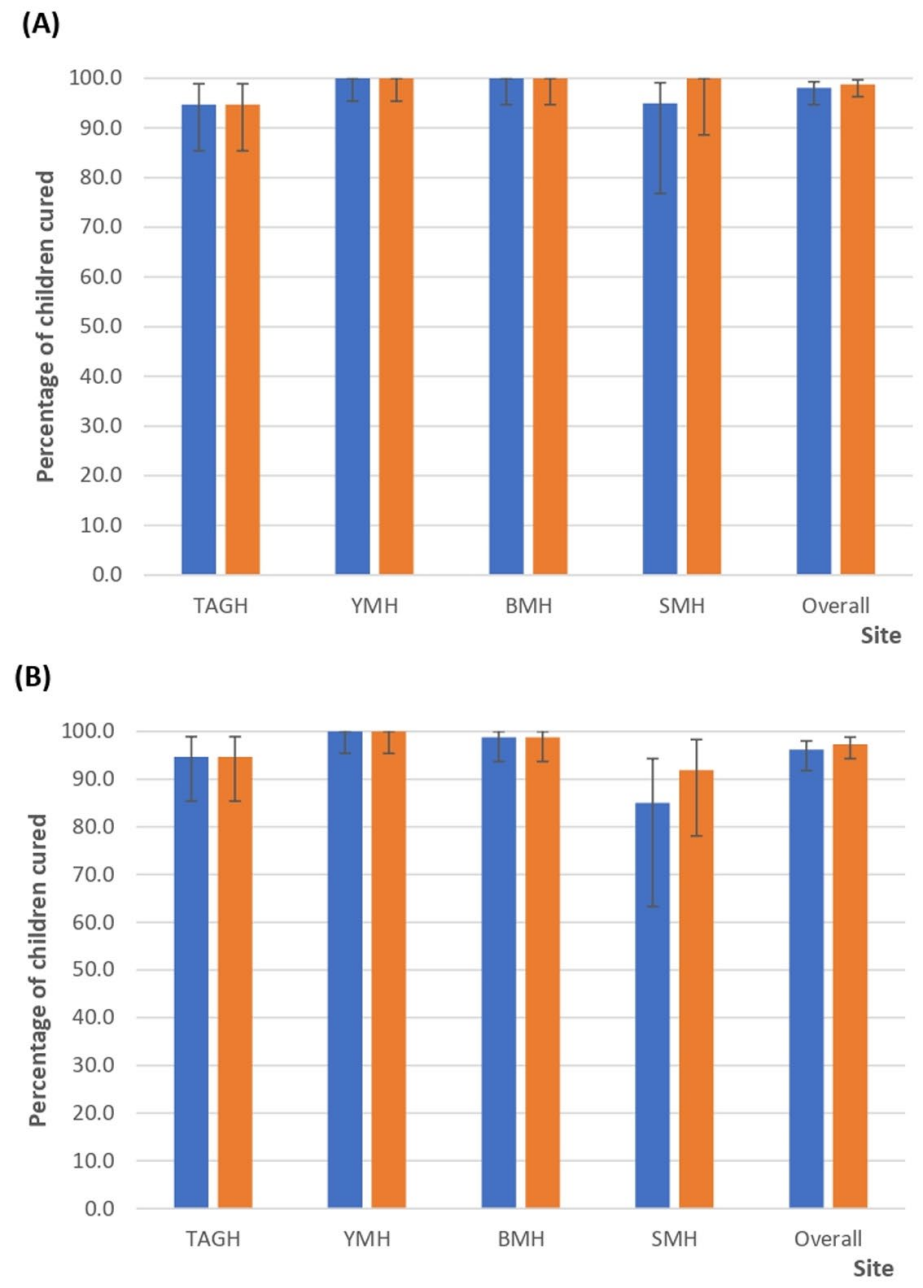
**(A)** Per protocol PCR-uncorrected and PCR-corrected cure rates on Day 28 following treatment with artesunate-pyronaridine (AP). Blue bars are PCR-uncorrected cure rates whilst orange bars are PCR-corrected cure rates. TAGH, Tarkwa Apinto Government Hospital; YMH, Yendi Municipal Hospital; BMH, Bekwai Municipal Hospital; SMH, Sunyani Municipal Hospital. **(B)** Per protocol PCR-uncorrected and PCR-corrected cure rates on Day 42 following treatment with artesunate-pyronaridine (AP). Blue bars are PCR-uncorrected cure rates whilst orange bars are PCR-corrected cure rates. TAGH, Tarkwa Apinto Government Hospital; YMH, Yendi Municipal Hospital; BMH, Bekwai Municipal Hospital; SMH, Sunyani Municipal Hospital.

PCR-uncorrected cure rates on day 28 were 100% in two of the four sites that studied AP: YMH and BMH. The three treatment failures occurring within 28 days of follow-up in TAGH were all confirmed as recrudescence whilst the two treatment failures in SMH were both confirmed as re-infections yielding Day-28 PCR-corrected cure rates of 94.7% (95% CI: 85.4–98.9) in TAGH and 100% in SMH with an overall PCR-corrected cure rate of 98.8% (95% CI: 96.3–99.7) (Figure 5A). PCR-uncorrected cure rates on Day-42, following treatment with AP, were 100% in YMH, 94.7% (95% CI: 85.4–98.9) in TAGH, 98.8% (95% CI: 93.7–100) in BMH, and 85.0% (95% CI: 70.2–94.3) in SMH yielding an overall PCR-uncorrected cure rate of 96.2% (95% CI: 92.9–98.0). All three treatment failures identified during the 42-day follow-up period in TAGH were confirmed to be recrudescence; the only treatment failure in BMH was also confirmed to be recrudescence; whilst three of the six failures in SMH were confirmed to be recrudescence, yielding Day-42 cure rates of 94.7% (95% CI: 85.4–98.9) in TAGH, 98.8% (95% CI: 93.7–100) in BMH, 91.9% (95% CI: 78.1–98.3) in SMH, and an overall PCR-corrected cure rate of 97.3% (95% CI: 94.3–98.8) (Figure 5B).

**Figure 6:**
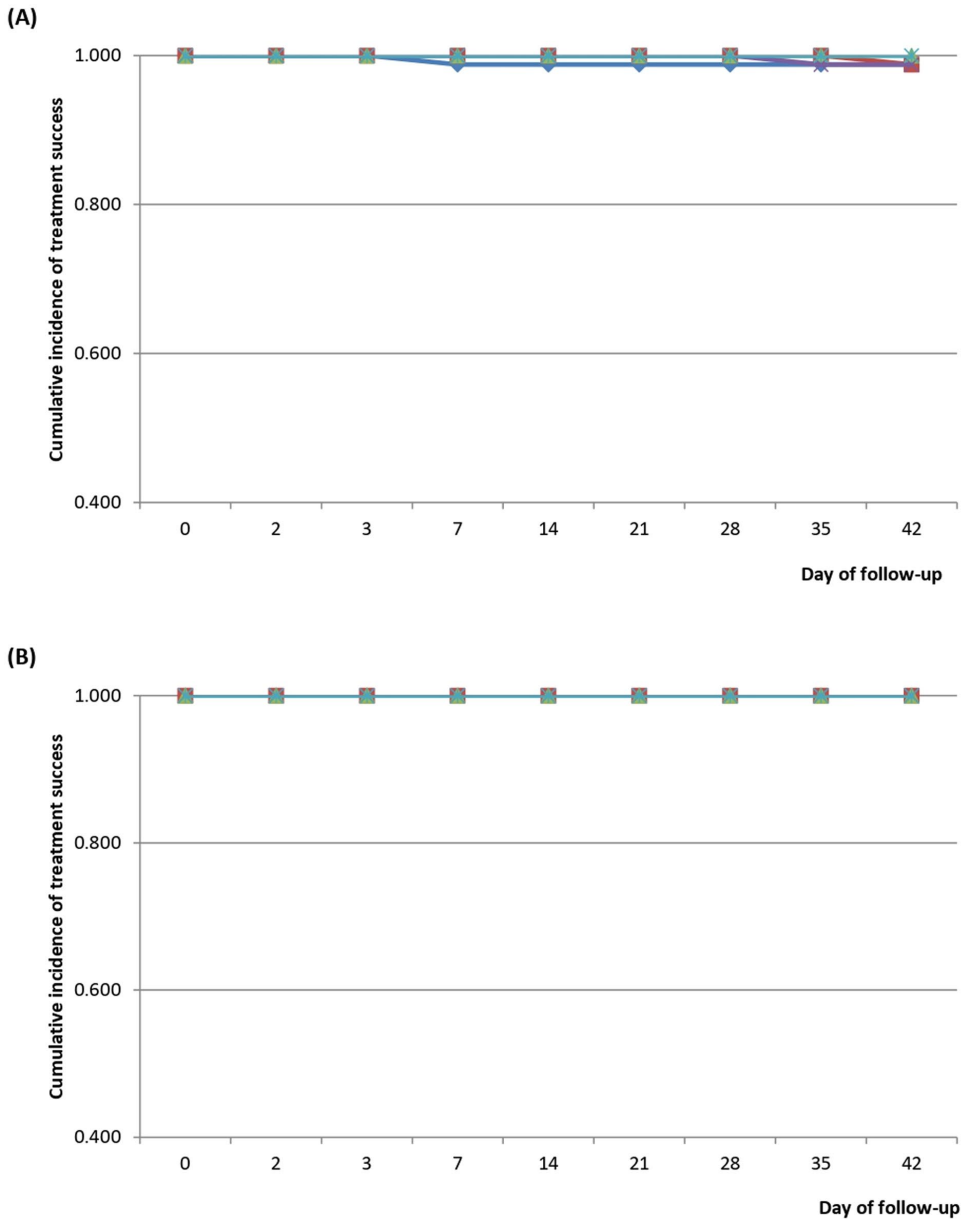
**(A)** PCR-uncorrected Kaplan–Meier survival curve for children treated with dihydroartemisinin-piperaquine (DHAP). Blue line represents Wa Urban Health Centre (WUHC); red line represents Navrongo War Memorial Hospital (NWMH); light green line represents Volta Regional Hospital (VRH); purple line represents Begoro Government Hospital (BGH); and light blue represents Ewim Polyclinic (EWP). **(B)** PCR-corrected Kaplan–Meier survival curve for children treated with dihydroartemisinin-piperaquine (DHAP). Blue line represents Wa Urban Health Centre (WUHC); red line represents Navrongo War Memorial Hospital (NWMH); light green line represents Volta Regional Hospital (VRH); purple line represents Begoro Government Hospital (BGH); and light blue represents Ewim Polyclinic (EWP).

Kaplan–Meier survival analyses of patients treated with DHAP showed cumulative incidence of PCR-uncorrected treatment success of 1.000 for all sites between Day 0 and Day 3. This dropped to 0.988 (95% CI: 0.925–0.999) on Day 7 in WUHC, and remained same between Day 7 and Day 42. The drop in BGH occurred on Day 35 (0.988; 95% CI: 0.924–0.999), and was same on Day 42. The drop in NWMH occurred on Day 42 (0.988; 95% CI: 0.924–0.999). The other two DHAP sites (VRH and EWP) showed a cumulative incidence of PCR-uncorrected treatment success of 1.000 throughout the 42-day follow-up period (Figure 6A). Incidence of PCR-corrected treatment success remained 1.000 for all DHAP sites throughout the 42-day follow-up period (Figure 6B).

**Figure 7:**
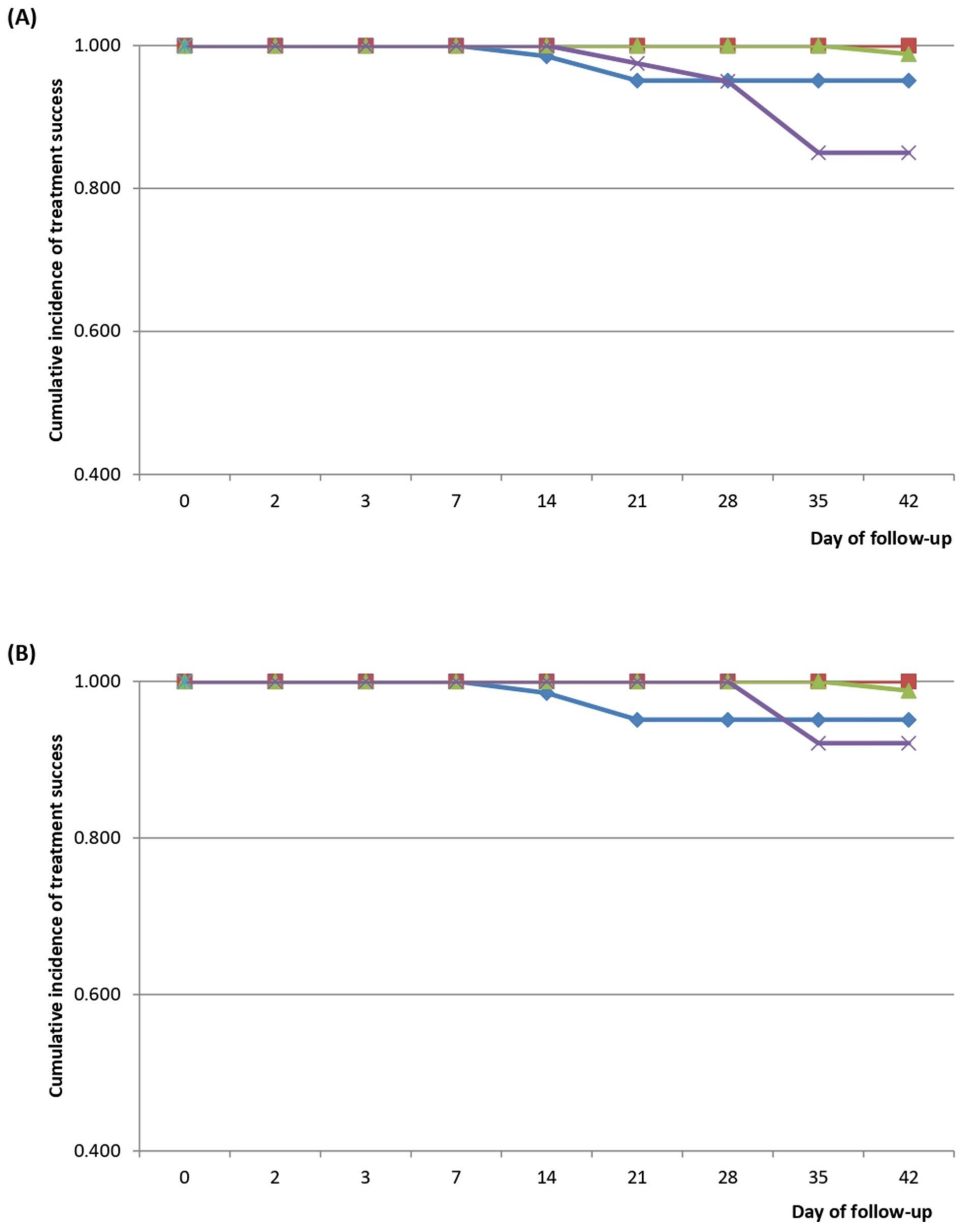
**(A)** PCR-uncorrected Kaplan–Meier survival curve for children treated with artesunate-pyronaridine (AP). Blue line represents Tarkwa Apinto Government Hospital (TAGH); red line represents Yendi Municipal Hospital (YMH); light green line represents Bekwai Municipal Hospital (BMH); and purple line represents Sunyani Municipal Hospital (SMH). **(B)** PCR-corrected Kaplan–Meier survival curve for children treated with artesunate-pyronaridine (AP). Blue line represents Tarkwa Apinto Government Hospital (TAGH); red line represents Yendi Municipal Hospital (YMH); light green line represents Bekwai Municipal Hospital (BMH); and purple line represents Sunyani Municipal Hospital (SMH).

Kaplan–Meier survival analyses of patients treated with AP showed cumulative incidence of PCR-uncorrected treatment success of 1.000 for all sites between Day 0 and Day 7. This dropped to 0.985 (95% CI: 0.898–1.000) on Day 14 in TAGH, and further dropped to 0.951 (95% CI: 0.856–0.985) on Day 21 but remained same between Day 21 and Day 42. The drop in BMH (0.988; 95% CI: 0.920–0.998) occurred on Day 42 whilst the drop in SMH (0.975; 95% CI: 0.853–0.999) occurred on Day 21. This further dropped to 0.950 (95% CI: 0.818–0.991) on Day 28, 0.850 (95% CI: 0.696–0.930) on Day 35 and remained same on Day 42 (Figure 7A). Cumulative incidence of PCR-corrected treatment success followed similar patterns in TAGH, YMH, and BMH as observed with PCR-uncorrected treatment success (Figure 7B). However, the drop in SMH (0.921; 95% CI: 0.775–0.974) occurred on Day 35 and not Day 21 as observed with PCR-uncorrected treatment success in the site, and this remained same on Day 42 (Figure 7B).

### 3.3 Secondary study outcomes

The prevalence of measured fever (axillary temperature ≥ 37.5 °C), among children treated with DHAP, significantly decreased from Day 0 to Day 1 in all five sites: 76.3% (95% CI: 65.2–84.8) to 16.3% (95% CI: 9.3–26.6) in BGH; 83.6% (95% CI: 71.5–91.4) to 8.3% (95% CI: 3.1–19.1) in EWP; 98.8% (95% CI: 92.7–99.9) to 0.0% in VRH; 70.2% (95% CI: 59.1–79.4) to 4.9% (1.6–12.7) in WUHC; and 91.8% (95% CI: 83.3–96.4) to 3.6% (95% CI: 0.9–10.9) in NWMH. Prevalence of measured fever on Day 2 was 0.0% in three sites: EWP, VRH, and NWMH; 1.3% (95% CI: 0.1–7.8) in BGH; and 1.2% (95% CI: 0.1–7.5) in WUHC. None of the children had an axillary temperature ≥ 37.5 °C on Day 7 (Supplementary Figure S1A). Similarly, prevalence of measured fever, among children treated with AP, significantly decreased from Day 0 to Day 1 in all four sites: 90.8% (95% CI: 80.4–96.2) to 0.0% in TAGH; 90.7% (95% CI: 82.0–95.6) to 17.4% (95% CI: 10.4–27.4) in BMH; 90.5% (95% CI: 76.5–96.9) to 17.5% (95% CI: 7.9–33.4) in SMH; and 11.5% (95% CI: 5.7–21.2) to 0.0% in YMH. None of the children had an axillary temperature ≥ 37.5 °C on Days 2, 3, and 7 (Supplementary Figure S1B).

Parasitological assessment for the first week of follow-up in DHAP sites showed two children with parasites on Day 2: one child in WUHC and another child in NWMH. There was only one child in EWP with parasites on Day 3, and another child in WUHC with parasites on Day 7 (Supplementary Figure S2A). The child with parasites on Day 7 was confirmed by PCR-genotyping to have a re-infection (Supplementary Table S1). Parasitological assessment in AP sites showed three children with parasites on Day 2: two in TAGH and one in SMH whilst only one site (TAGH) had a parasitaemic child on Day 3. There was no child with parasites in all four AP sites on Day 7 (Supplementary Figure S2B). Gametocytes were present on Day 0 (pre-treatment) in five children in the DHAP sites: one in BGH, two in EWP, and two in NWMH. By Day 14, there was only one child in VRH with gametocytes. No child in the DHAP sites was gametocytemic on Days 21, 28, 35, and 42 (Supplementary Figure S3A). Gametocytes were present in two children in the AP sites: one in BMH and the other in SMH. No child in the AP sites had gametocytes on Days 7, 14, 21, 28 and 42 (Supplementary Figure S3B).

Mean haemoglobin levels significantly increased in all DHAP sites during the 28-day follow-up period whilst three of the AP sites showed significant increases. However, extending follow-up period to 42 days yielded a significant increase in all sites, irrespective of treatment given (Supplementary Table S4).

The main adverse event was vomiting. A total of 63 children in the DHAP group (16.0%; 95% CI: 12.6–20.1) vomited at least once during the 3 days of treatment whilst 33 children in the AP group (12.2%; 95% CI: 8.7–16.8) vomited at least once within the same period (*p* = 0.209). None of the children who vomited in either of the two groups failed treatment.

## 4 Discussion

The therapeutic efficacy of DHAP and AP was studied in sentinel sites across Ghana with the objective of generating follow-up data on the efficacy of DHAP (since the last study in 2020/2021) (10) as well as the efficacy of AP, which was a newly introduced first-line ACT for the treatment of uncomplicated malaria in the country (7). This discussion covers main findings related to the primary and secondary outcomes of the study conducted between August and December 2023.

The study showed that the overall prevalence of parasitaemia on Day 3, which is critical in the assessment of partial artemisinin resistance, is 0.3% for DHAP and 0.4% for AP. The children who were parasitaemic on Day 3 showed adequate clinical and parasitological responses on Day 28 and Day 42. The observed parasite prevalence levels on day 3 are far below the WHO’s threshold of 10% for partial artemisinin resistance (14), suggesting that the artemisinin component of ACT continues to be effective in the treatment of uncomplicated malaria in Ghana. These findings compare well with those from a couple of previous studies conducted in Ghana, Nigeria, Togo, Ethiopia, and Grande Comore Island between 2020 and 2022 (10, 15–18).

The study also showed all five DHAP sites with PCR-corrected cure rates of 100% on Day 28. This finding compares well with that of the 2020 study, signalling that PCR-corrected cure rate of DHAP assessed on Day 28 has not changed (10). The overall PCR-corrected cure rate on Day 42 was 100% compared with the previous rate of 97.0% (95% CI: 93.4–98.8%) (*p* = 0.003). The significant difference between the two time points could be attributed to the different methodology for PCR-correction. Whereas the current study used three markers (*msp1, msp2, and glurp*), the previous study used only one marker (*msp2*) leading to a probable over-estimation of treatment failure in the previous study (19). Indeed, NWMH with a previous PCR-corrected cure rate of 90.3% (95% CI: 79.5–96.0) – the least at the time using only *msp2* (10) showed a PCR-corrected cure rate of 100% in the current study when all three markers were used for the PCR analysis. This finding, therefore, suggests that DHAP remains highly efficacious in the treatment of uncomplicated malaria in Ghana as in neighbouring Togo (16) and other African countries such as Mozambique (20) and Uganda (21).

For AP, the study showed an overall Day-28 PCR-corrected cure rate of 98.8% (95% CI: 96.3–99.7), which compares well with the 97.4% (95% CI: 90.2–99.6) observed in Nigeria (15). Even though the Nigeria study ended on Day 28, our overall Day-42 PCR-corrected cure rate of 97.3% (95% CI: 94.3–98.8) compares well with the 98.9% (95% CI: 92.2–99.8) observed in Ethiopia (17), and 97.8% (95% CI: 94.1–100) observed in Mozambique (20) establishing a baseline data that suggests that AP is highly efficacious in the treatment of uncomplicated malaria in Ghana.

With regard to fever and parasite clearance, both DHAP and AP effected rapid clearance in all sites. No child was febrile by the second day following commencement of treatment (Day 2) whilst prevalence of parasitaemia on day 3 was less than 1% for both ACTs. These findings compare well with several studies in Africa (10, 15–17, 20, 22–24). Similarly, haemoglobin levels significantly increased in all sites during the 42-day follow-up period suggestive of the role of DHAP and AP treatments in improving the haemoglobin levels of uncomplicated malaria patients (10, 20).

This study has three main limitations. First, pharmacokinetic analyses of partner drugs (piperaquine and pyronaridine) were not done to help distinguish between treatment failures due to suboptimal drug levels and failures due to drug resistant parasites. The seemingly lower efficacy levels of AP, compared with DHAP (97.3% versus 100%), is therefore inconclusive, coupled with the fact that this was not a comparative study. Second, the use of *glurp* in the 3/3 approach to distinguish between reinfection and recrudescence could underestimate recrudescence because of the limited discriminating nature of *glurp* (13). Additionally, two of the three DHAP failures and six of the 10 AP failures did not have all markers amplified on day of failure. This may have overestimated recrudescence (Supplementary Table S1). This notwithstanding, overall PCR-uncorrected cure rates for DHAP and AP were 99.2% (95% CI: 97.6–99.8) and 96.2% (95% CI: 92.9–98.0), respectively. Subsequent studies will employ microsatellite, which is more discriminatory than *glurp* (in addition to *msp1* and *msp2*) (13), whilst exploring funding and capacity for amplicon sequencing, which is the most robust and reliable genotyping method (13). Third, the small sample size (about half of the required sample size) of the study in SMH (*N* = 42) makes the site-specific day-42 PCR-corrected cure rate of 91.9% (95% CI: 78.1–98.3) less reliable as evidenced by the wide confidence interval.

## 5 Conclusion

We conclude that the therapeutic efficacy level of DHAP has remained high in Ghana (> 90%) since the baseline study in 2020/2021. Also, the baseline efficacy level of AP is high (> 90%) warranting the use of both DHAP and AP in the treatment of uncomplicated malaria in the country. Coupled with this, is the fact that there is no current indication of partial artemisinin resistance in the country based on day-3 parasitaemia of less than 1%.

## Data Availability

The original contributions presented in the study are included in the article/Supplementary material. Further inquiries can be directed to the corresponding author.
