# Supplementary figures and images for: Therapeutic efficacy of dihydroartemisinin-piperaquine and artesunate-pyronaridine combinations in the treatment of uncomplicated Plasmodium falciparum malaria in Ghana, 2023
Source: Front Public Health. 2026 Jan 5;13:1715777. doi: 10.3389/fpubh.2025.1715777 (PMC12821887; doi:10.3389/fpubh.2025.1715777)

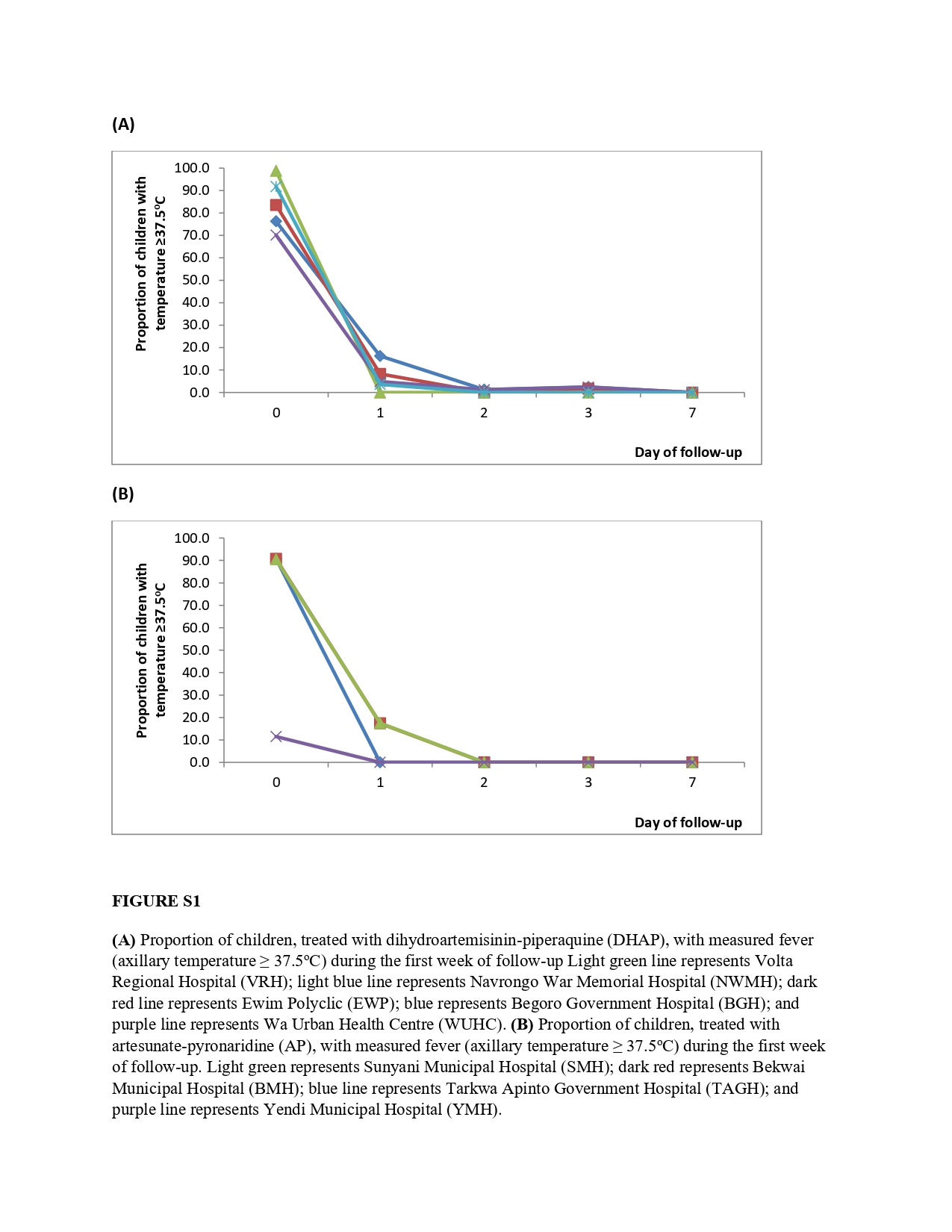

Supplement: Supplementary file 1 [file Image_1.tiff]

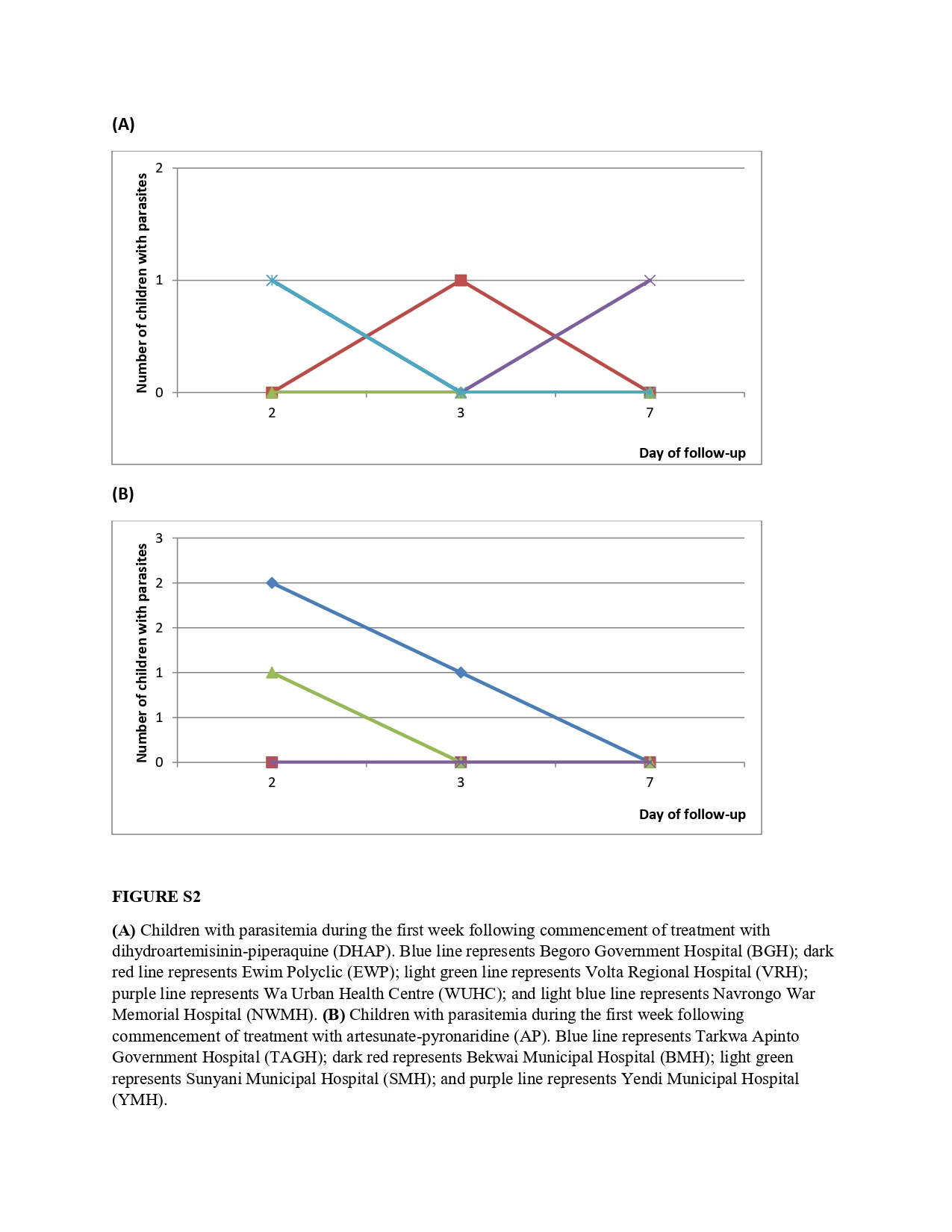

Supplement: Supplementary file 2 [file Image_2.tiff]

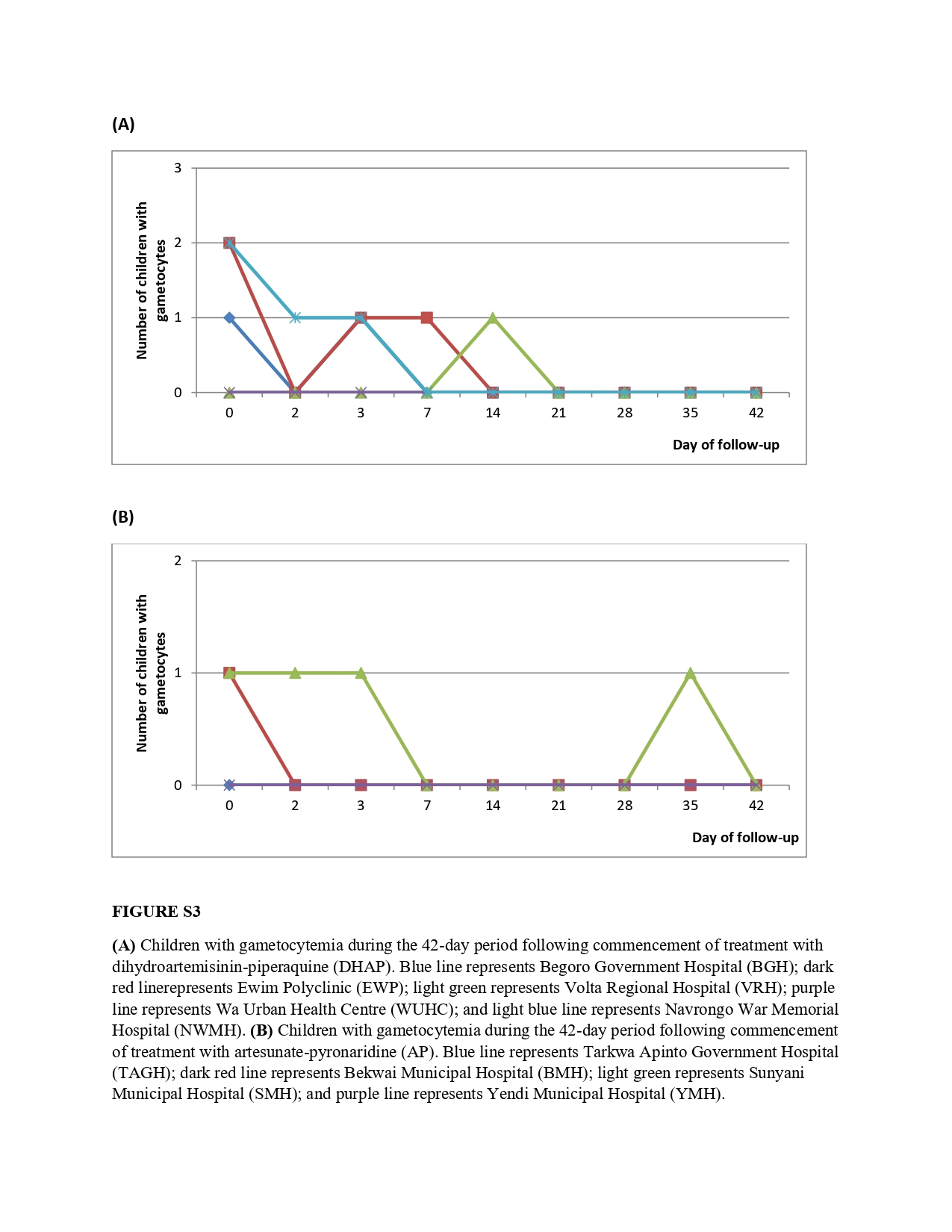

Supplement: Supplementary file 3 [file Image_3.tiff]
