# Supplementary material for: Therapeutic efficacy of dihydroartemisinin-piperaquine and artesunate-pyronaridine combinations in the treatment of uncomplicated Plasmodium falciparum malaria in Ghana, 2023
Source: Front Public Health. 2026 Jan 5;13:1715777. doi: 10.3389/fpubh.2025.1715777 (PMC12821887; doi:10.3389/fpubh.2025.1715777)
Supplement: Supplementary file 4 [file Table_1.docx]

Table S1 Outcome of PCR genotyping of treatment failures using *msp1, msp2* and *glurp*

| **SAMPLE ID** | **SITE** | **TYPE OF ACT** | **MSP1** | | | **MSP2** | | **GLURP** | **REMARKS** | |
| --- | --- | --- | --- | --- | --- | --- | --- | --- | --- | --- |
|  |  |  | **RO33 (bp)** | **MAD20 (bp)** | **K1 (bp)** | **IC 3D7 (bp)** | **FC (bp)** | **GLURP (bp)** | **3/3 Approach** | **msp1+msp2** |
| B138- Day 0 | BMH | AP | 200,300,600 | _- | 300,400,500 | - | 300,**400**,500 | **500**,800,1000 |  |  |
| B138- Day 42 |  |  | _- | _- | _- | _- | **400** | **500** | Recrudescence | Recrudescence |
|  |  |  |  |  |  |  |  |  |  |  |
| G045- Day 0 | BGH | DHAP | 300,500 | 200 | 250,300,350,400 | 700 | **300,400** | 700,900 |  |  |
| G045- Day 35 |  |  | _- | _- | _- | _- | **300,400** | 500,1250,1500 | Reinfection | Recrudescence |
|  |  |  |  |  |  |  |  |  |  |  |
| N063-Day 0 | NWMH | DHAP | 300,100 | 200,300,400,1000 | 900,1000 | 200,**300**,500,**600** | 300,400,600 | 800,900 |  |  |
| N063-Day 42 |  |  | _- | _- | 250,300 | 250,**300,600** | 250,390 | 1000 | Reinfection | Reinfection |
|  |  |  |  |  |  |  |  |  |  |  |
| S021-Day 0 | SMH | AP | **200,300**,500,900 | 1000 | **250,300**,900,1000 | 400,600,700 | 290 | 800, 900, 1200 |  |  |
| S021-Day 28 |  |  | **200,300** | 200 | **250,300** | _- | 300 | _- | Reinfection | Reinfection |
|  |  |  |  |  |  |  |  |  |  |  |
| S025-Day 0 | SMH | AP | **200,300** | **200,300,500**,900 | _ | 400,**600,700,800** | 300 | 800,**900,1200** |  |  |
| S025 -Day 35 |  |  | **200,300** | **200,300,500** | 300,500 | 300,**600,700,800** | 400 | **900,1200** | Recrudescence | Recrudescence |
|  |  |  |  |  |  |  |  |  |  |  |
| S060- Day 0 | SMH | AP | 300,400 | 150,300,400 | 300 | 400,600,700,800 | 600,700 | 700 |  |  |
| S060 -Day 21 |  |  | _- | _- | _- | _- | _- | 450,550 | Reinfection | Indeterminate |
|  |  |  |  |  |  |  |  |  |  |  |
| S067- Day 0 | SMH | AP | **200,300,**550 | **200**,1000 | 250,300 | 400,600,700 | 600 | 1000,1200 |  |  |
| S067 -Day 35 |  |  | **200,300,**500 | **200**,300 | 400,500 | _- | 300 | _- | Reinfection | Reinfection |
|  |  |  |  |  |  |  |  |  |  |  |
| S085- Day 0 | SMH | AP | **200,300,**600 | _- | _- | **400,600,700** | 600 | _- |  |  |
| S085-Day 35 |  |  | **200,300** | _- | _- | 300,**400,600,700** | 300 | 750,900 | Recrudescence | Recrudescence |
|  |  |  |  |  |  |  |  |  |  |  |
| S117-Day 0 | SMH | AP | **400,1000** | **200**,300 | 400,500 | **600**,700 | _- | **1000,1200** |  |  |
| S117-Day 35 |  |  | 300,**400**,500,600,**1000** | 150,**200**,400,900 | _ | 500,**600** | 300 | **1000,1200** | Recrudescence | Recrudescence |
|  |  |  |  |  |  |  |  |  |  |  |
| T006-Day 0 | TAGH | AP | _- | _- | _- | **300**,400,600,700 | _- | 900 |  |  |
| T006-Day 21 |  |  | _- | -_ | -_ | 200,**300**,500 | _- | -_ | Recrudescence | Recrudescence |
|  |  |  |  |  |  |  |  |  |  |  |
| T070-Day 0 | TAGH | AP | - | 200 | **400** | **300**,400,**600**,**700**,800 | - | **800,900** |  |  |
| T070-Day 21 |  |  | - | -_ | **400**,500 | **300,600,700** | 400 | **800,900** | Recrudescence | Recrudescence |
|  |  |  |  |  |  |  |  |  |  |  |
| T092-Day 0 | TAGH | AP | _- | -_ | 400 | 500,600,1000 | 300 | 250,**500**,1000,1200 |  |  |
| T092-Day 21 |  |  | - | -_ | -_ | -_ | -_ | **500** | Recrudescence | Indeterminate |
|  |  |  |  |  |  |  |  |  |  |  |
| W022-Day 0 | WUHC | DHAP | 400 | - | 900,1000 | 300,400,500,600,700 | 290 | 800,1000,1200 |  |  |
| W022-Day 7 |  |  | 200 | -_ | -_ | -_ | -_ | -_ | Reinfection | Reinfection |
