# Supplementary material for: Therapeutic efficacy of dihydroartemisinin-piperaquine and artesunate-pyronaridine combinations in the treatment of uncomplicated Plasmodium falciparum malaria in Ghana, 2023
Source: Front Public Health. 2026 Jan 5;13:1715777. doi: 10.3389/fpubh.2025.1715777 (PMC12821887; doi:10.3389/fpubh.2025.1715777)
Supplement: Supplementary file 5 [file Table_2.docx]

TABLE S2 Per protocol DHAP treatment outcomes on Day 42

| **Treatment outcome** | **WUHC (N=84)** | **NWMH (N=85)** | **VRH (N=85)** | **BGH (N=80)** | **EWP (N=61)** | **Total (N=395)** |
| --- | --- | --- | --- | --- | --- | --- |
| **PCR-uncorrected**  **(Day 42)** |  |  |  |  |  |  |
| ETF  n (%, 95% CI) | 0 | 0 | 0 | 0 | 0 | 0 |
| LPF  n (%, 95% CI) | 1 (1.2, 0.0-6.6) | 1 (1.2, 0.0-6.7) | 0 | 1 (1.3, 0.0-6.8) | 0 | 3 (0.8, 0.2-2.4) |
| LCF  n (%, 95% CI) | 0 | 0 | 0 | 0 | 0 | 0 |
| ACPR  n (%, 95% CI) | 81 (98.8, 93.4-100) | 80 (98.8, 93.3-100) | 85 (100, 95.8-100) | 79 (98.8, 93.2-100) | 60 (100, 94.0-100) | 385 (99.2, 97.6-99.8) |
| Total per protocol | 82 | 81 | 85 | 80 | 60 | 388 |
| Lost/withdrawn  n (%, 95% CI) | 2 (2.4, 0.4-9.1) | 4 (4.7, 1.5-12.3) | 0 | 0 | 1 (1.6, 0.1-100) | 7 (1.8, 0.8-3.8) |
| **PCR-corrected**  **(Day 42)** |  |  |  |  |  |  |
| ETF  n (%, 95% CI) | 0 | 0 | 0 | 0 | 0 | 0 |
| LPF  n (%, 95% CI) | 0 | 0 | 0 | 0 | 0 | 0 |
| LCF  n (%, 95% CI) | 0 | 0 | 0 | 0 | 0 | 0 |
| ACPR  n (%, 95% CI) | 81 (100, 95.5-100) | 80 (100, 95.5-100) | 85 (100, 95.8-100) | 79 (100, 95.4-100) | 60 (100, 94.0-100) | 385 (100, 98.8-100) |
| Total per protocol | 81 | 80 | 85 | 79 | 60 | 385 |
| Lost/withdrawn,  n (%, 95% CI) | 3 (3.6, 0.9-10.8) | 5 (5.9, 2.2-13.8) | 0 | 1 (1.3, 0.1-7.7) | 1 (1.3, 0.1-7.7) | 10 (2.5, 1.3-4.8) |

*DHAP* Dihydroartemisinin-piperaquine, *ETF* Early Treatment Failure, *LPF* Late Parasitological Failure, LCF Late Clinical Failure, *ACPR* Adequate Clinical and Parasitological Response, *WUHC* Wa Urban Health Centre, *NWMH* Navrongo War Memorial Hospital, *VRH* Volta Regional Hospital, *BGH* Begoro Government Hospital, *EWP* Ewim Polyclinic
