# Supplementary material for: Therapeutic efficacy of dihydroartemisinin-piperaquine and artesunate-pyronaridine combinations in the treatment of uncomplicated Plasmodium falciparum malaria in Ghana, 2023
Source: Front Public Health. 2026 Jan 5;13:1715777. doi: 10.3389/fpubh.2025.1715777 (PMC12821887; doi:10.3389/fpubh.2025.1715777)
Supplement: Supplementary file 6 [file Table_3.docx]

TABLE S3 Per protocol AP treatment outcomes on Day 42

| **Treatment outcome** | **TAGH (N=65)** | **YMH (N=78)** | **BMH (N=86)** | **SMH (N=42)** | **Total (N=271)** |
| --- | --- | --- | --- | --- | --- |
| **PCR-uncorrected**  **(Day 42)** |  |  |  |  |  |
| ETF  n (%, 95% CI) | 0 | 0 | 0 | 0 | 0 |
| LPF  n (%, 95% CI) | 3 (5.3, 1.1-14.6) | 0 | 1 (1.2, 0.0-6.3) | 6 (15.0, 5.7-29.8) | 10 (3.8, 2.0-7.2) |
| LCF  n (%, 95% CI) | 0 | 0 | 0 | 0 | 0 |
| ACPR  n (%, 95% CI) | 54 (94.7, 85.4-98.9) | 78 (100, 95.4-100) | 85 (98.8, 93.7-100) | 34 (85.0, 70.2-94.3) | 251 (96.2, 92.9-98.0) |
| Total per protocol | 57 | 78 | 86 | 40 | 261 |
| Lost/withdrawn  n (%, 95% CI) | 8 (12.3, 5.8-23.4) | 0 | 0 | 2 (4.8, 0.8-17.4) | 10 (3.7, 1.9-6.9) |
| **PCR-corrected**  **(Day 42)** |  |  |  |  |  |
| ETF  n (%, 95% CI) | 0 | 0 | 0 | 0 | 0 |
| LPF  n (%, 95% CI) | 3 (5.3, 1.1-14.6) | 0 | 1 (1.2, 0.0-6.3) | 3 (8.1, 1.7-21.9) | 7 (2.7, 1.2-5.8) |
| LCF  n (%, 95% CI) | 0 | 0 | 0 | 0 | 0 |
| ACPR  n (%, 95% CI) | 54 (94.7, 85.4-98.9) | 78 (100, 95.4-100) | 85 (98.8, 93.7-100) | 34 (91.9, 78.1-98.3) | 251 (97.3, 94.3-98.8) |
| Total per protocol | 57 | 78 | 86 | 37 | 258 |
| Lost/withdrawn,  n (%, 95% CI) | 8 (12.3, 5.8-23.4) | 0 | 0 | 5 (11.9, 4.5-26.4) | 13 (4.8, 2.7-8.3) |

*AP* Artesunate-pyronaridine, *ETF* Early Treatment Failure, *LPF* Late Parasitological Failure, LCF Late Clinical Failure, *ACPR* Adequate Clinical and Parasitological Response, *TAGH* Tarkwa Apinto Government Hospital, *YMH* Yendi Municipal Hospital, *BMH* Bekwai Municipal Hospital, *SMH* Sunyani Municipal Hospital
