# Supplementary material for: Therapeutic efficacy of dihydroartemisinin-piperaquine and artesunate-pyronaridine combinations in the treatment of uncomplicated Plasmodium falciparum malaria in Ghana, 2023
Source: Front Public Health. 2026 Jan 5;13:1715777. doi: 10.3389/fpubh.2025.1715777 (PMC12821887; doi:10.3389/fpubh.2025.1715777)
Supplement: Supplementary file 7 [file Table_4.docx]

Table S4 Changes in mean hemoglobin levels following treatment with DHAP and AP

| Day | Mean Hb (sd) by ACT and site | | | | | | | | |
| --- | --- | --- | --- | --- | --- | --- | --- | --- | --- |
|  | DHAP | | | | | AP | | | |
|  | BGH | EWP | VRH | WUHC | NWMH | TAGH | BMH | SMH | YMH |
| Day 0 | 10.6 (1.3)  N=80 | 9.3 (1.7)  N=61 | 10.7 (1.5)  N=85 | 10.1 (1.5)  N=83 | 10.2 (1.4)  N=85 | 10.4 (1.6)  N=65 | 10.6 (1.1)  N=86 | 10.6 (1.5)  N=42 | 10.6 (0.9)  N=78 |
| Day 28 | 11.0 (1.0)  N=80 | 11.0 (1.0)  N=60 | 11.2 (1.2)  N=85 | 11.0 (1.0)  N=83 | 11.5 (0.8)  N=80 | 10.8 (1.4)  N=58 | 11.2 (0.9)  N=86 | 11.3 (1.3)  N=40 | 10.9 (0.7)  N=78 |
| Day 42 | 11.2 (1.0)  N=80 | 11.4 (0.9)  N=60 | 11.4 (1.2)  N=85 | 11.5 (0.9)  N=82 | 12.1 (0.6)  N=80 | 11.5 (1.3)  N=53 | 11.4 (0.9)  N=86 | 11.9 (1.1)  N=37 | n/d |
| *p-value*  *(Day 0 vs. Day 28)* | 0.031 | 0.000 | 0.018 | 0.000 | 0.000 | 0.145 | 0.000 | 0.027 | 0.021 |
| *p-value*  *(Day 0 vs. Day 42)* | 0.002 | 0.000 | 0.001 | 0.000 | 0.000 | 0.001 | 0.000 | 0.000 | n/a |

*DHAP* Dihydroartemisinin-piperaquine, *AP* Artesunate-pyronaridine; *sd* standard deviation, *WUHC* Wa Urban Health Centre, *NWMH* Navrongo War Memorial Hospital, *VRH* Volta Regional Hospital, *BGH* Begoro Government Hospital, *EWP* Ewim Polyclinic, *TAGH* Tarkwa Apinto Government Hospital, *YMH* Yendi Municipal Hospital, *BMH* Bekwai Municipal Hospital, *SMH* Sunyani Municipal Hospital, *n/d* not done, *n/a* not applicable
